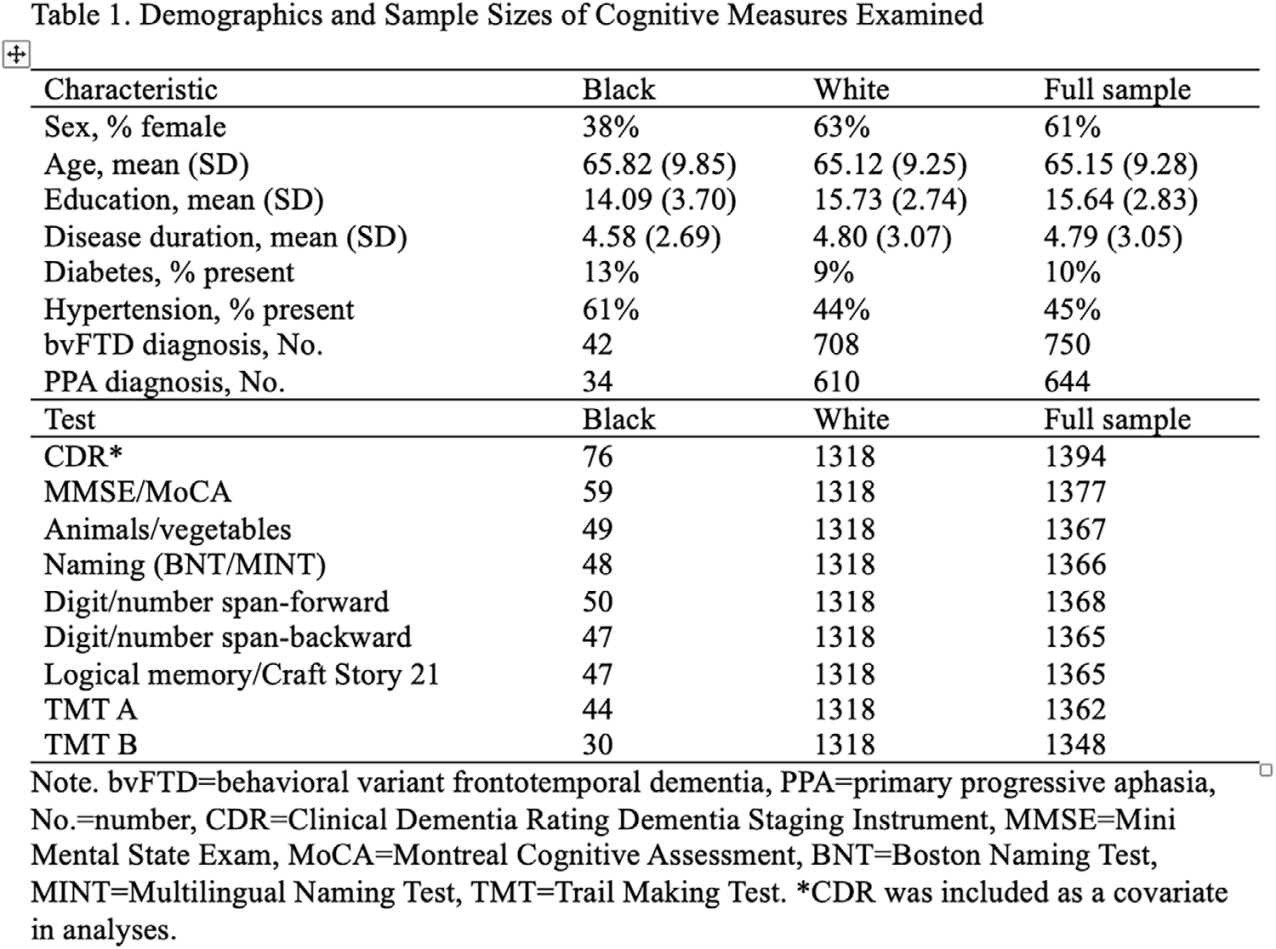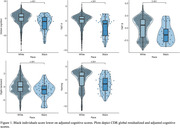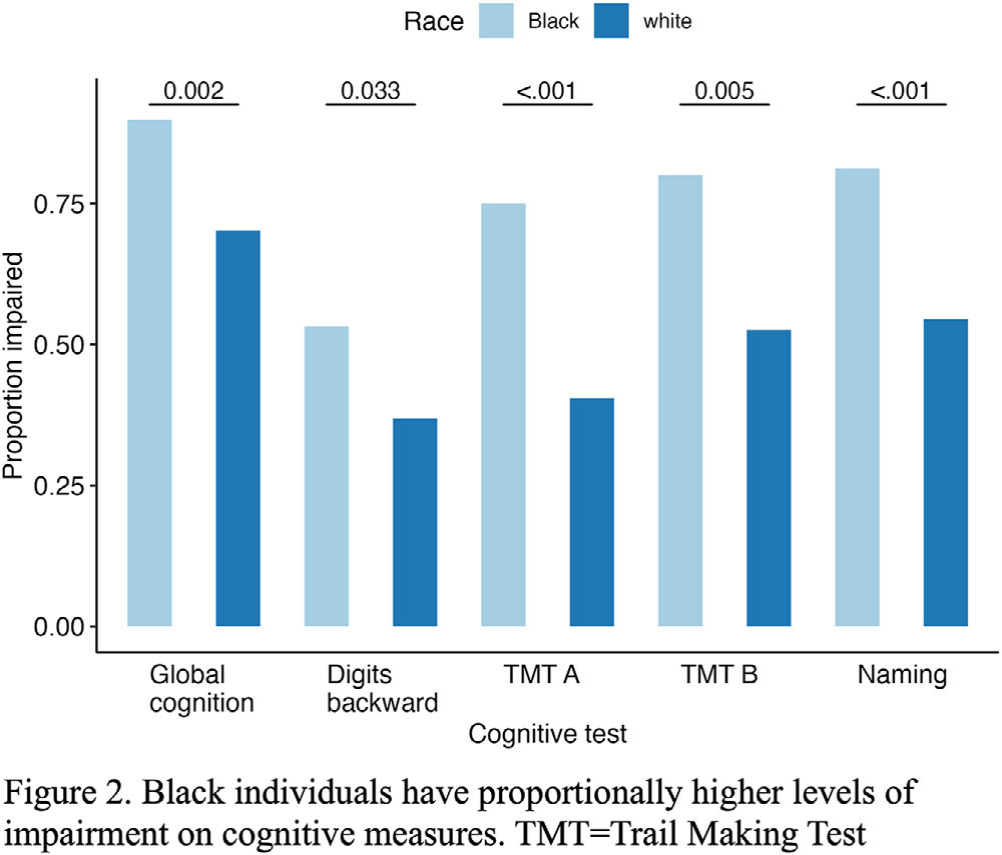# Evaluation of neuropsychological test performance between minoritized and White individuals with frontotemporal degeneration

**DOI:** 10.1002/alz.095515

**Published:** 2025-01-09

**Authors:** Melanie A Matyi, Emma Rhodes, Sheina Emrani, Hannah A Jin, David J Irwin, Corey T McMillan, Lauren Massimo

**Affiliations:** ^1^ Penn Frontotemporal Degeneration Center, Department of Neurology, Perelman School of Medicine, University of Pennsylvania, Philadelphia, PA USA

## Abstract

**Background:**

Minoritized groups are an understudied population in frontotemporal degeneration (FTD) research. Recently, we demonstrated distinct neuropsychiatric symptom profiles in Black relative to White individuals with FTD but cognition across these groups has not been reported. This knowledge gap has potential implications for the care of individuals with FTD from minoritized groups. Here we evaluated neuropsychological performance across Black and White individuals with FTD using the National Alzheimer’s Coordinating Center (NACC) Uniform Dataset (UDS).

**Methods:**

Individuals with a behavioral variant FTD or primary progressive aphasia diagnosis, neuropsychological data, and self‐identified as non‐Hispanic White (n = 1318) or Black (n = 30‐76) were included. Data from UDS versions (1‐3) were adjusted for age, sex, and education using established norms. Linear regressions examined the association between race and normative cognitive scores (see Table 1 for measures and sample sizes), covarying for CDR‐Global. Chi‐square tests then examined group differences in the proportion of participants classified as impaired (≤‐1.5 normative score).

**Results:**

Black individuals on average had lower scores (Fig. 1) and greater likelihood of impairment (Fig. 2) on measures of global cognition (β = ‐3.07; χ^2^ = 9.65), digits backward (β = ‐.624; χ^2^ = 4.53), trails A (β = ‐2.22; χ^2^ = 19.54) and B (β = ‐1.20; χ^2^ = 7.79), and naming (β = ‐1.79; χ^2^ = 12.4). We did not identify any neuropsychological tests where White individuals performed worse than Black individuals.

**Conclusions:**

Neuropsychological data in representative populations is severely lacking in the NACC cohort diagnosed with FTD, comprised of only 3% Black individuals and 7% other minoritized groups. We observed that on average Black individuals have reduced performance relative to their White counterparts on several cognitive domains. Importantly, performance differences translated to the classification of a greater proportion of Black, compared to White, individuals as impaired, in part because available norms do not account for race. Of note, our ability to i) explore differences in cognition across minoritized groups and ii) assess for differences in cognitive decline was hampered by the limited sample of participants from minoritized groups with available data. Future efforts must focus on increasing research participation in underrepresented populations with FTD to evaluate potential sources of the observed differences across racial groups and support the diverse needs of all individuals.